# Modulation of the Effect of Cisplatin on Nicotine-Stimulated A549 Lung Cancer Cells Using Analog of Marine Sponge Toxin Loaded in Gelatin Nanoparticles

**DOI:** 10.3390/nano14090777

**Published:** 2024-04-30

**Authors:** Ahmad Joukhan, Veno Kononenko, Marija Sollner Dolenc, Matej Hočevar, Tom Turk, Damjana Drobne

**Affiliations:** 1 Department of Pharmaceutical Chemistry, Faculty of Pharmacy, University of Ljubljana, 1000 Ljubljana, Slovenia; marija.sollner@ffa.uni-lj.si; 2 Department of Biology, Faculty of Biotechnical, University of Ljubljana, 1000 Ljubljana, Slovenia; veno.kononenko@bf.uni-lj.si (V.K.); tom.turk@bf.uni-lj.si (T.T.); 3 Institute of Metals and Technology, 1000 Ljubljana, Slovenia; matej.hocevar@imt.si

**Keywords:** marine toxin, nAChR, nAChR antagonist, APS12-2, chemotherapeutic agent

## Abstract

Nicotine activates nicotinic acetylcholine receptors (nAChRs), which are overexpressed in numerous cancer types, leading to signaling pathways that increase lung cancer invasiveness and resistance to chemotherapeutic agents. In this study, the effects of APS12-2, a synthetic analog of marine sponge toxin that acts as an antagonist of nAChRs, was investigated in vitro on A549 human lung adenocarcinoma cells and non-tumorigenic human lung epithelial BEAS-2B cells. In addition, gelatin nanoparticles (GNPs) loaded with APS12-2 (APS12-2-GNPs) were prepared and their effects were compared with those of free APS12-2. Nicotine reduced cytotoxicity, the formation of reactive oxygen species, and the formation of lipid droplets caused by cisplatin on A549 cells. The effects of nicotine on the decreased efficacy of cisplatin were reduced by APS12-2 and APS12-2-GNPs. APS12-2-GNPs showed a substantial advantage compared with free APS12-2; the cytotoxicity of APS12-2 on BEAS-2B cells was greatly reduced when APS12-2 was loaded in GNPs, whereas the cytotoxicity on A549 cells was only slightly reduced. Our results suggest that both APS12-2 and APS12-2-GNPs hold promise as supportive agents in the cisplatin-based chemotherapy of lung cancer.

## 1. Introduction

Lung cancer represents a global health crisis, as it is the second most commonly diagnosed cancer and the leading cause of cancer death worldwide [1]. Lung cancer is primarily categorized into two histological types—small-cell lung cancer (SCLC) and non-small-cell lung cancer (NSCLC). NSCLC accounts for approximately 85% of all lung cancer cases, while the remaining 15% are attributed to SCLC [2]. The treatment of NSCLC is a major challenge, as it often becomes resistant to chemotherapeutic treatments [2], and conventional chemotherapy alone proves ineffective in patients with advanced NSCLC [3]. One of the biggest challenges in cancer treatment with chemotherapy is the indiscriminate action of the drug on both malignant and healthy cells [4], which leads to side effects in other tissues and organs. In addition, cancer cells often develop resistance to chemotherapy, causing treatments to become less effective [4]. This leads to the administration of higher doses to overcome cancer resistance, resulting in more damage to healthy tissues.

The development of effective targeted therapies and immunotherapies for lung cancer has significantly improved the overall survival rate of patients with NSCLC [3,5,6]. Targeted therapies can use different antagonists that target specific genes and proteins or to change the microenvironment of tumors that promote the growth and survival of cancer cells [5]. Targeted therapies are frequently employed in treating advanced lung cancers that remain unaffected by conventional drugs [5]. In this context, it is interesting to investigate the modulation of nicotinic acetylcholine receptors (nAChRs), which are overexpressed in numerous cancer types [7].

nAChRs are ion channels located in the plasma membrane of the cells. They consist of five subunits that assemble into either heteropentameric receptors, comprising combinations of different subunits such as α2–α6 or α10 with β2–β4, or homopentameric receptors composed of five identical α7, α8, or α9 subunits [8]. Specific nAChR subtypes are selectively overexpressed in various cancer types. For example, α7 nAChRs are overexpressed in lung cancer, while α9 nAChRs show elevated levels in breast cancer [9].

Nicotine, a known nAChR agonist and the addictive component of tobacco, cannot trigger tumorigenesis, but it is known to promote tumor growth and metastasis [10]. Nicotine binds to and activates the nAChRs overexpressed in numerous cancer types, leading to the activation of various signaling pathways that increase lung cancer invasiveness and resistance to chemotherapeutics [11]. The stimulation of α7 nAChRs in lung cancer cells was associated with increased cellular growth and metastasis [12], as well as cancer resistance to chemotherapeutics [11]. According to the role of nAChRs in lung cancer cell development and drug resistance, the use of nAChR antagonists could block, or at least attenuate, the effect of nicotine (and other agonists, e.g., acetylcholine), thereby improving the efficacy of current chemotherapy agents such as cisplatin in combating cancer resistance.

Cisplatin is a versatile anticancer drug that is effective against various types of cancer and has been shown to have the potential to prolong patient survival [13]. The cytotoxicity of cisplatin has been shown to be related to the generation of reactive oxygen species (ROS) [14]. ROS are highly reactive molecules with unpaired electrons, such as superoxide, hydrogen peroxide, and hydroxyl radicals [15]. Cisplatin-induced ROS can elevate lipid peroxidation and alter enzymes and structural proteins, leading to cell apoptosis [16]. Despite its common use in the treatment of lung cancer, cisplatin often encounters resistance; nevertheless, it remains a primary therapy for lung cancer [17]. Therefore, there is a need to explore novel treatment approaches that improve the effectiveness of current therapies in eliminating cancer cells, while minimizing side effects and combating cancer resistance.

**Figure 1:**
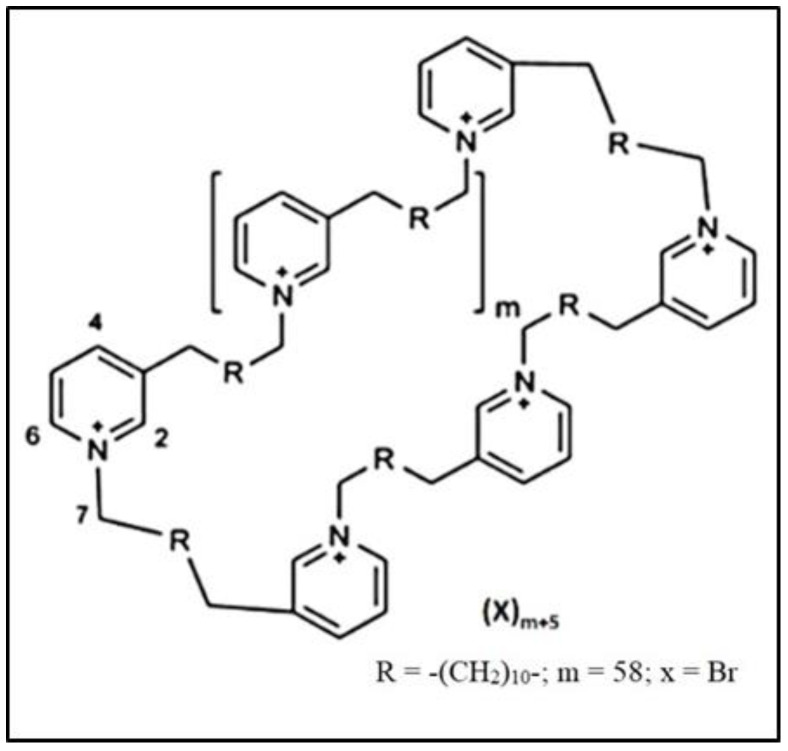
The chemical structure of 3-alkylpyridinium analog APS12-2.

So far, only a limited number of nAChR antagonists have been tested to examine their anticancer activities. For instance, sinomenine has been shown to decrease the proliferation and migration of lung cancer cells and trigger apoptosis [18]. Sinomenine is extracted from the roots of a plant called Sinomenium acutum [19]. Witayateeraporn et al. (2020) found that low doses of QND7, a chemically synthesized nAChR ligand, decreased the proliferation and migration of lung cancer cells [20]. Another example of a synthesized nAChR ligand, MG624, was found to inhibit angiogenesis in human SCLC [21]. Marine organisms have proven to be a valuable source of compounds with antitumor activities [7]. For example, polymeric 3-alkylpyridinium salts (poly-APSs), derived from the marine sponge *Haliclona* (*Rhizoneira*) *Sarai*, demonstrated inhibitory effects on the proliferation of NSCLC [22]. Poly-APSs exhibit various biological activities, including cytotoxicity, inhibition of bacterial growth, and cholinesterase activity inhibition [7]. Synthetic poly-APS analogs such as APS8 (3-octylpyridinium) and APS12-2 (1,3-dodecylpyridinium salt; Figure 1), obtained via organic synthesis, offer promising commercial and biological applications [23]. APS8 has been proven to be a potent α7 nAChR antagonist that counteracts the anti-apoptotic effects of nicotine and suppresses lung cancer cell growth, with negligible effects on normal lung fibroblasts [7]. Another synthetic poly-APS analog is APS8-2 [24]; our previous study showed that APS8-2 acts as an α7 nAChR antagonist and is able to counteract the pro-cancer effects of nicotine and restore the efficacy of cisplatin [25]. APS12-2 and structurally related compounds are described as potential chemotherapeutic agents that exhibit very low toxicity in vivo [26]. Grandič et al. (2012) showed that APS12-2 is a potent antagonist of skeletal muscle α12β1γδ nAChR [27]. Due to its structural similarity to APS8 and APS8-2, APS12-2 may also act as an antagonist for the α7 nAChR subtype and modulate the effects of nicotine that reduce the efficacy of chemotherapeutics. However, the use of different APS molecules in cancer therapy may be limited, due to their cytotoxic properties, which can negatively affect healthy cells.

Nanoparticles (NPs) offer several advantages in the administration of drugs. These include targeted delivery, which minimizes the drug’s effect on non-target cells; protection against degradation; improved tissue penetration; and the ability to incorporate several different substances into the same particle [28,29]. Gelatin nanoparticles (GNPs), which are biodegradable, non-toxic, and cost-effective, represent a promising tool for drug delivery [30]. GNPs have already been used clinically for the aerosolic delivery of drugs for the treatment of lung cancer, facilitating direct drug delivery into the lungs and potentially reducing side effects [31].

The aim of this study was to investigate the potential of APS12-2 and APS-12-2 loaded in GNPs (APS12-2-GNPs) to reduce the effects of nicotine, which decreases the efficacy of the chemotherapeutic agent cisplatin. The cytotoxicity of APS-12-2 and APS12-2-GNPs was tested on A549 non-small-cell lung cancer cells and BEAS-2B non-tumorigenic lung cells. The efficiency of APS12-2-GNPs to reduce the effect of nicotine on cisplatin-treated A549 cells was tested and compared with the effect of free APS-12-2.

## 2. Materials and Methods

### 2.1. Chemicals

APS12-2 (1,3-dodecylpyridinium salt) was obtained from Prof. Michael Jaspars from the University of Abeerden, Scotland, where it was synthesized [24]. A549 cells and BEAS-2B cells were obtained from American Type Culture Collection (ATCC, Manassas, VA, USA). The ApoTox-Glo^TM^ Triplex assay was obtained from Promega (Madison, WI, USA). Cell culture media, bovine serum albumin (BSA), phosphate-buffered saline (PBS), Dragendorff reagent, Gelatin type B (bloom 225), Nile red, and all other chemicals used in our experiments were obtained from Sigma-Aldrich (Steinheim, Germany), unless stated otherwise.

### 2.2. Preparation of Gelatin NPs

GNPs and APS12-2-GNPs were prepared using the nanoprecipitation technique, as described by Khan and Schneider (2013) [32], with modifications. Briefly, 20 mg of gelatin type B (bloom 225) was dissolved in 1 mL of distilled water, to prepare free GNPs. For the preparation of APS12-2-GNPs, 20 mg of gelatin type B (bloom 225) was dissolved in a water solution containing 5 mg/mL APS12-2. In the following steps, the gelatin–water solution was heated to 55 °C and was slowly added (drop by drop) to a solution consisting of 95% ethanol and 7% (*m*/*v*) Poloxamer 407, using a magnetic stirrer for mixing. Cross-linking of the gelatin solution was achieved by adding a 2% glutaraldehyde solution. The solution was left overnight, while being continuously mixed with a magnetic stirrer. The synthesized GNPs and APS12-2-GNPs were freeze-dried. The mass of the synthesized particles was determined after freeze-drying. Prior to the experiments, the dried GNPs and APS12-2-GNPs were rehydrated with Milli-Q water.

### 2.3. Characterization of Gelatin NPs

GNPs and APS12-2-GNPs were visualized using scanning electron microscopy (SEM; JEOL JSM-6500F, JEOL, Akishima, Tokyo, Japan) and transmission electron microscopy (TEM; JEM 2100, JEOL, Akishima, Tokyo, Japan). Zeta potential measurements were performed on the GNPs and APS12-2-GNPs suspended in cell medium and water, using electrokinetic measurements (Brookhaven Instruments Corporation, ZetaPALS, Holtsville, NY, USA). The hydrodynamic diameter of GNPs and APS12-2-GNPs in water was determined using dynamic light scattering (DLS; Litesizer 500, Anton Paar, Graz, Austria).

### 2.4. APS12-2 Loading and Profile Release

The concentration of APS12-2 loaded in APS12-2-GNPs was measured using Dragendorff reagent, a colorimetric reagent commonly used for detecting alkaloid content [33,34]. Standard calibration curves were created with varying concentrations of APS12-2 (0, 1, 1.5, 2, and 2.5 µg/mL). In a 96-well plate, 100 µL of different concentrations of APS12-2, along with solutions containing 50 µg/mL of GNPs and 50 µg/mL of APS12-2-GNPs, were pipetted. Subsequently, 10 µL of Dragendorff reagent was added to each well, while a blank containing 110 µL of distilled water (without Dragendorff reagent) was prepared. The plate was briefly agitated to ensure homogeneity and the absorbance was measured at 300 nanometers (nm) using a BioTek Cytation 3 spectrophotometer. The amount of APS12-2 in the APS12-2-GNPs was determined using the calibration curve. This experiment was conducted in duplicate, with each instance involving five replicates.

The release profile of APS12-2 from prepared GNPs was studied in phosphate-buffered saline (PBS) at pH 7.4 and 37 °C, using a Wisestir MSH-20D stirrer (witeg Labortechnik GmbH, Wertheim, Germany) at 100 rpm. A total of 50 µg/mL APS12-2-GNPs were prepared in PBS and were allowed to incubate for various time intervals (0.5, 1, 2, 4, 6, 24, and 48 h). At each time point, 2 mL of the suspension was pipetted and centrifuged at 14,000× *g* for 5 min; subsequently, the supernatant was transferred into a new Falcon tube. The absorbance of the supernatant at each time interval was measured using a BioTek Cytation 3 spectrophotometer (Seattle, WA, USA). The amount of released APS12-2 in the supernatant was quantified, using a calibration curve previously generated for APS12-2.

### 2.5. Cell Culture

A549 cells and BEAS-2B cells were cultured in Dulbecco’s modified Eagle’s medium, supplemented with 4 mM L-glutamine and 10% (*v*/*v*) fetal bovine serum (FBS). Cells were grown at 37 °C in a humidified atmosphere with 5% CO_2_. For the experiments with BEAS-2B cells, flasks/plates coated with collagen type I and BSA were used.

### 2.6. Cytotoxicity Measurements

The cytotoxicity of APS12-2, GNPs, and APS12-2-GNPs was assessed on A549 lung cancer cells and the non-cancerous lung epithelial cell line BEAS-2B. Three different assays, namely the Resazurin assay (RUZ), the Neutral red uptake (NRU) assay, and the Coomassie brilliant blue (CBB) assay, were used to measure cytotoxicity, each providing a unique perspective on cell viability. The RUZ assay measures cellular metabolic activity, the NRU assay measures the stability of lysosomes in live cells, and the CBB assay measures the quantity of cellular proteins. A549 and BEAS-2B cells were seeded in 96-well plates at a density of 7000 and 10,000 cells per well, respectively. After a 24 h incubation period to allow for cell adhesion, cells were treated with different concentrations of APS12-2 (from 0.18 to 1.8 µg/mL), GNPs (from 10 to 400 µg/mL), and GNPs-APS12-2 (from 10 to 200 µg/mL) for 24 h. After exposure, cytotoxicity assays were performed, according to the protocol described by Kononenko and Drobne [35]. For all experiments, three independent repeats were performed, each with at least five replicates.

### 2.7. ApoTox-Glo^TM^ Triplex Assay

A549 cells were seeded in 96-well plates at a density of 7000 cells per well and were incubated for 24 h. The cells were then treated with different compounds for 24 h or 48 h. The cells were treated for 24 h with 1 µM nicotine; 50 µg/mL cisplatin; 0.18 µg/mL APS12-2; 10 µg/mL GNPs; 10 µg/mL APS12-2-GNPs (equivalent to 0.18 µg/mL APS12-2); a combination of 1 µM nicotine and 50 µg/mL cisplatin; a combination of APS12-2, 50 µg/mL cisplatin, and 1 µM nicotine; and a combination of APS12-2-GNPs, 1 µM nicotine, and 50 µg/mL cisplatin. The cells were treated for 48 h with 1 µM nicotine; 10 µg/mL cisplatin; 0.18 µg/mL APS12-2; 10 µg/mL GNPs; 10 µg/mL APS12-2-GNPs (equivalent to 0.18 µg/mL APS12-2); a combination of 1 µM nicotine and 10 µg/mL cisplatin; a combination of APS12-2, 10 µg/mL cisplatin, and 1 µM nicotine; and a combination of APS12-2-GNPs, 1 µM nicotine, and 10 µg/mL cisplatin. After 24 h or 48 h of treatment, cytotoxicity was assessed using the ApoTox-Glo^TM^ Triplex Assay, following the manufacturer’s instructions (Promega, Madison, WI, USA). Four replicates were performed for each treatment condition.

### 2.8. Intracellular ROS Measurements

A549 cells were seeded in 96-well plates at a density of 7000 cells per well and were incubated for 24 h to allow for cell adhesion. The cells were then treated for 24 h with 1 µM nicotine, 10 µg/mL GNPs, 10 µg/mL APS12-2-GNPs (equivalent to 0.18 µg/mL APS12-2), 0.18 µg/mL APS12-2, and a combination of these compounds with 1 µM nicotine. After the treatment, cells were rinsed with 100 µL of PBS. The cells were then incubated with 20 µM 2′,7′-dichlorofluorescein diacetate (DCFH-DA) for 30 min, to load the ROS indicator, followed by two PBS rinses to remove unloaded dye. Finally, the cells were treated with 100 µg/mL Cisplatin and then 7′-dichlorofluorescein (DCF) fluorescence was measured using a BioTek Cytation 3 microplate reader (Seattle, WA, USA), at an excitation wavelength of 488 nm and an emission wavelength of 520 nm, to assess ROS levels inside the cells. This compound transforms into the highly fluorescent DCF as a result of oxidation induced by ROS. For each treatment condition, at least three independent repetitions were performed.

### 2.9. Lipid Droplet Measurements

A549 cells were seeded in 12-well plates at a density of 80,000 cells per well and incubated for 24 h to allow cell adhesion. Cells were then treated for 24 h with 1 µM nicotine; 50 µg/mL cisplatin; a combination of nicotine and cisplatin; 10 µg/mL GNPs; 0.18 µg/mL APS12-2; a combination of APS12-2, cisplatin, and nicotine; 10 µg/mL APS12-2-GNPs (equivalent to 0.18 µg/mL APS12-2); or a combination of APS12-2-GNPs, nicotine, and cisplatin. The next day, the treatment was removed and cells were washed with 100 µL PBS. The cells were then incubated with 1 µM Nile red for 10 min. The fluorescence intensities of at least 10,000 cells per sample were analyzed, using a flow cytometer (BD FACSMelody™) equipped with a yellow-green laser and filter (BP/613/18LP/605/10). Data were analyzed using the FlowJo software V10 (FlowJo, Ashland, OR, USA). For each treatment condition, three independent repetitions were performed.

### 2.10. Statistical Analysis

The results are expressed as mean ± standard deviation (SD) and were statistically analyzed using ANOVA with Bonferroni’s post-test for multiple comparisons. Statistical significance was determined by a *p*-value below 0.05. Statistical analysis was performed using GraphPad Prism software 5 (GraphPad Software, San Diego, CA, USA).

## 3. Results

### 3.1. Nanoparticle Characteristics

**Figure 2:**
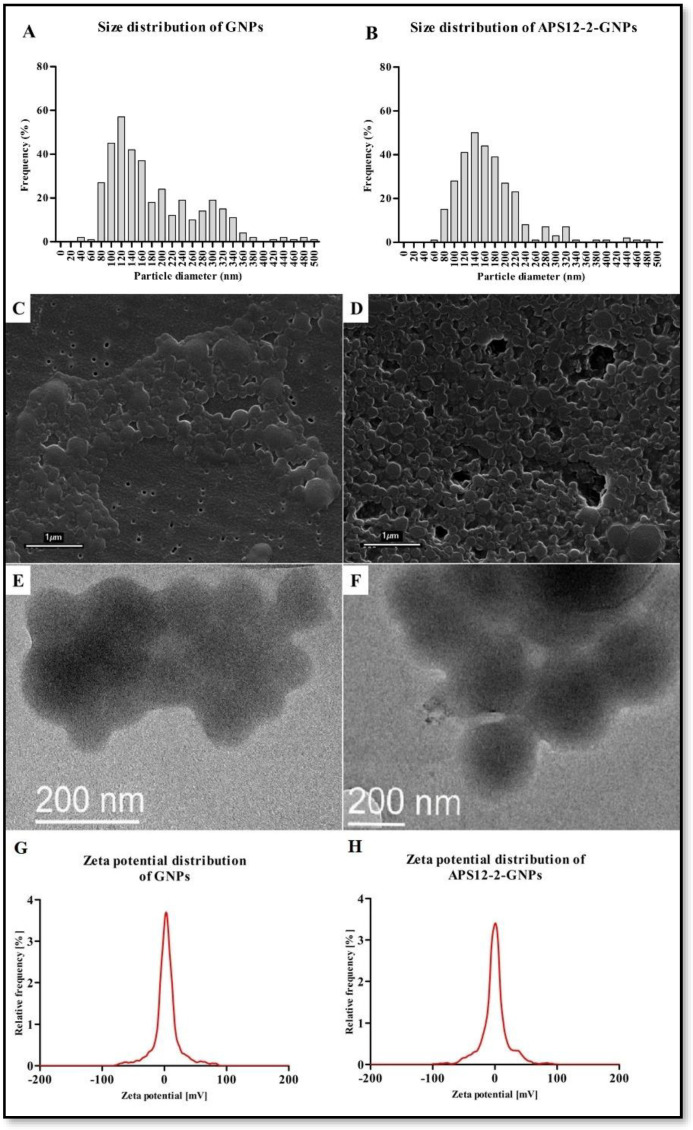
Size distribution, scanning electron microscopy (SEM) images, transmission electron microscopy (TEM) images, and zeta potential distribution in cell medium (pH 8.10) of GNPs and APS12-2GNPs. (**A**) The size distribution, (**C**) the scanning electron microscopy images, (**E**) the transmission electron microscopy image and (**G**) the zeta potential distribution of GNPs, and (**B**) the size distribution, (**D**) the scanning electron microscopy images, (**F**) the electron microscopy image, and (**H**) the zeta potential distribution in cell medium (pH 8.12) of APS12-2-GNPs.

The mean diameter of GNPs was 182 ± 87 nm, with median value of 185 nm (Figure 2A), while the mean diameter of APS12-2-GNPs was 167 ± 65 nm, with median value of 156 nm, with a broader size distribution (Figure 2B). The hydrodynamic diameter of GNPs and APS12-2-GNPs in water, as determined using DLS, was 316 ± 6 nm and 360 ± 22 nm, respectively. The zeta potential of GNPs in water and cell culture medium (Figure 2G) was −26.0 ± 0.7 mV and −6.2 ± 0.6 mV, respectively, while for APS12-2-GNPs (Figure 2H), was 8.3 ± 5.8 mV and −7.7 ± 1.7 mV, respectively.

### 3.2. Profile Release of APS12-2-GNPs

**Figure 3:**
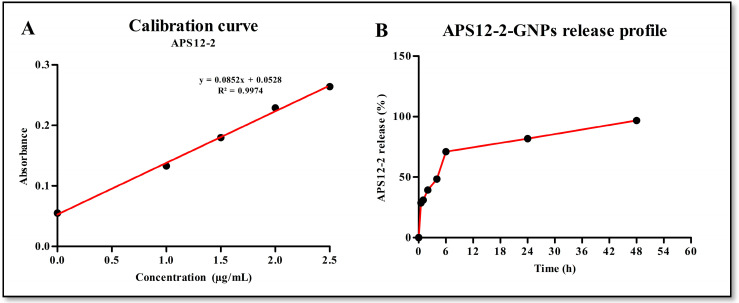
Calibration curve of APS12-2 (**A**) and release profile of APS12-2-GNPSs (**B**), n = 5.

The amount of APS12-2 within the APS12-2-GNPs was determined using Dragendorff reagent (Figure 3A). APS12-2-GNPs contained 0.90 ± 0.05 µg APS12-2 per 50 µg of APS12-2-GNPs, as determined using a calibration curve generated from various concentrations of free APS12-2 (Figure 3A). The APS12-2 released from the prepared GNPs exhibited a burst release pattern during the initial 6 h, with approximately 70% of APS12-2 being released within this period (Figure 3B). The release of APS12-2 followed a controlled and slower manner over the next 48 h. By the end of the 48 h, approximately 96% of the APS12-2 had been released (Figure 3B).

### 3.3. Cytotoxicity of APS12-2, GNPs, and APS12-2-GNPs

The cytotoxicity of APS12-2, APS12-2-GNPs, and GNPs was evaluated on A549 lung cancer cells and the non-cancerous lung epithelial cell line BEAS-2B, after 24 h exposure using three different assays—the Resazurin assay, the neutral red uptake (NRU) assay, and the Coomassie brilliant blue (CBB) assay (Figure 3). APS12-2 and APS12-2-GNPs induced concentration-dependent cytotoxicity in both cancerous (A549) and non-cancerous (BEAS-2B) lung cells (Figure 3), whereas GNPs did not induce cytotoxicity in either cell line (Figure 3A,B).

**Figure 4:**
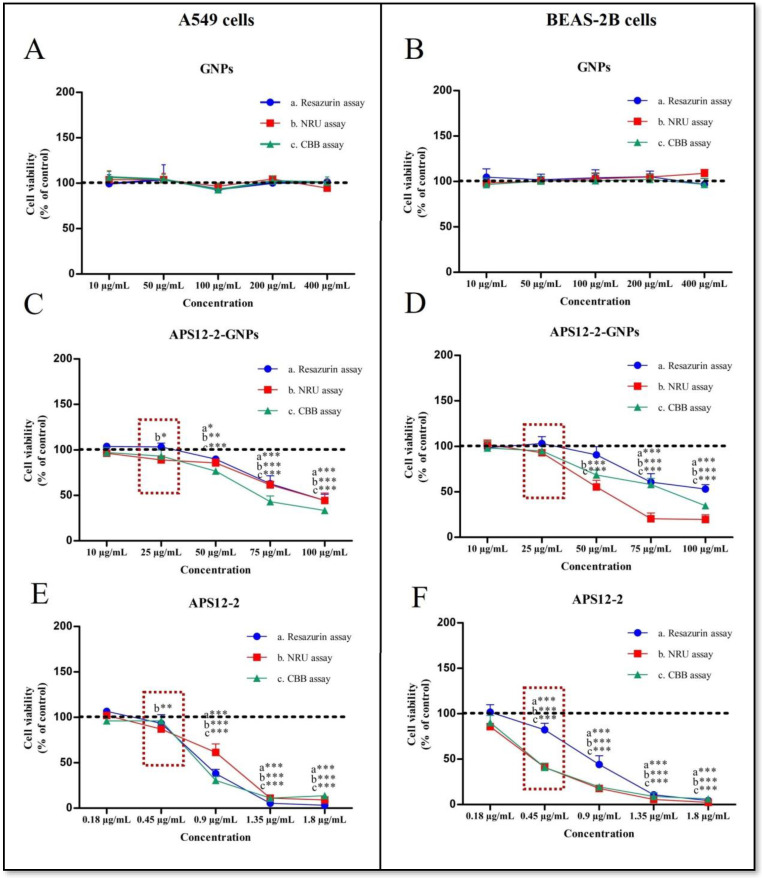
The cytotoxicity of APS12-2, GNPs, and APS12-2-GNPs on A549 and BEAS-2B cells. The cytotoxicity (**A**,**B**) GNPs, (**C**,**D**) APS12-2-GNPs, and (**E**,**F**) APS12-2 on (**A**,**C**,**D**) A549 cells and on (**B**,**D**,**F**) BEAS-2B cells was evaluated using the Resazurin, NRU, and CBB assays, after 24 h of exposure. Dashed rectangles represent the differences in the cytotoxicity of APS12-2 and APS12-2-GNPs in both cell lines. Measurements were normalized to untreated controls (dashed line), as mean percentage (+SD, n = 3). The data were statistically analyzed using ANOVA with Bonferroni multiple comparisons post-test. The asterisks indicate a significant difference with respect to the untreated control. * equals *p* < 0.05; ** equals *p* < 0.01; *** equals *p* < 0.001.

At a concentration of 0.45 µg/mL of APS12-2, there was a significant decrease in metabolic activity (measured using the Resazurin assay), lysosomal stability (NRU assay), and protein content (CB assay) in BEAS-2B cells (Figure 4F). However, in A549 cells, only a slight change was observed in lysosomal stability (NRU assay) at the same concentration. The cytotoxicity of APS12-2 decreased in both cell lines, when loaded into gelatin nanoparticles. Specifically, in BEAS-2B cells (Figure 4F), APS12-2 induced higher cytotoxicity than in A549 cells (Figure 4E). In A549 cells, we detected low, but significant, cytotoxicity (using the NRU assay) at 25 µg/mL APS12-2-GNPs (Figure 4C), which corresponds to 0.45 µg/mL APS12-2 (since 25 µg of APS12-2-GNPs contains 0.45 µg of APS12-2). In contrast, in BEAS-2B cells, no significant cytotoxicity was observed with APS12-2-GNPs at the same concentration (Figure 4D). At concentrations ≥50 µg/mL, APS12-2-GNPs exhibited cytotoxicity in both cell lines (Figure 4C,D).

In our other experiments, we used non-cytotoxic concentrations of APS12-2 (0.18 µg/mL) and APS12-2-GNPs (10 µg/mL).

### 3.4. Modulation of Cisplatin-Induced Cytotoxicity in A549 Cells by Nicotine, APS12-2, or APS12-2-GNPs

**Figure 5:**
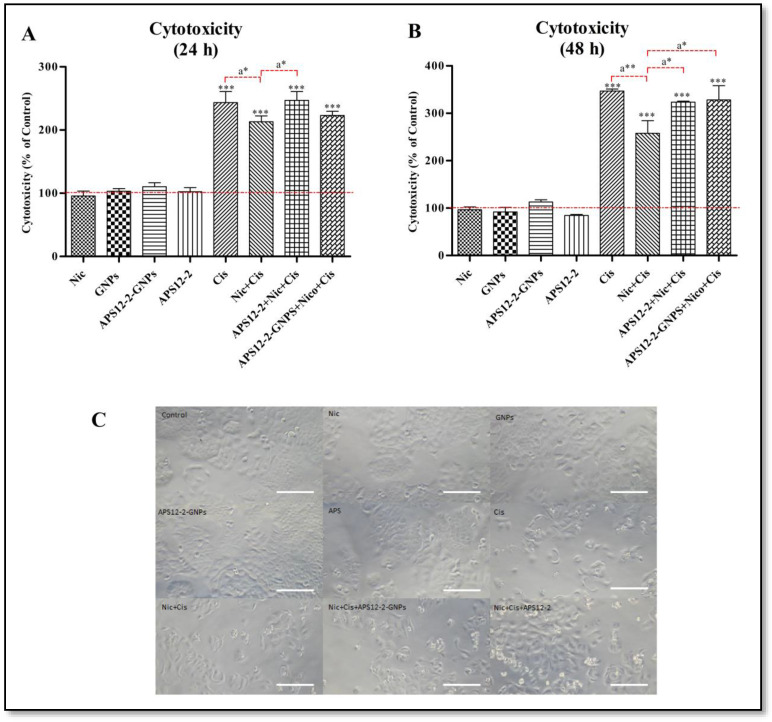
Measurement of cytotoxicity using the ApoTox-Glo^TM^ Triplex assay in A549 cells. Cytotoxicity was measured in A549 cells exposed to different compounds for (**A**) 24 h and (**B**) 48 h: (**A**,**B**) 1 µM nicotine (Nic), (**A**,**B**) 10 µg/mL GNPs, (**A**,**B**) 10 µg/mL APS12-2-GNPs (equivalent to 0.18 µg/mL APS12-2), (**A**,**B**) 0.18 µg/mL APS12-2, (**A**) 50 µg/mL cisplatin (Cis), (**B**) 10 µg/mL cisplatin (Cis), (**A**,**B**) a combination of Nic and Cis, (**A**,**B**) a combination of APS12-2, Cis, and Nic, and (**A**,**B**) a combination of APS12-2-GNPs, Nic, and Cis. (**C**) Microscopic images of A549 cells treated with 1 µM Nic; 10 µg/mL GNPs; 10 µg/mL APS12-2-GNPs; 0.18 µg/mL APS12-2; 50 µg/mL cis; a combination of Cis and Nic; a combination of APS12-2, Cis, and Nic; and a combination of APS12-2-GNPs, Nic, and Cis for 24 h; scale bar represents 100 μm. Measurements are normalized to the untreated controls and presented as mean percentage (+SD, n = 4). The dashed line represents the cytotoxicity of untreated control cells. The data were statistically analyzed using ANOVA with Bonferroni multiple comparisons post-test. The asterisks indicate a significant difference with respect to the untreated control. *** equals *p* < 0.001 and, with respect to cells treated with Nic and Cis, a* equals *p* < 0.05; a** equals *p* < 0.01.

The cytotoxicity of various compounds was evaluated on A549 cells after 24 h and 48 h treatments, using the ApoTox-Glo^TM^ Triplex assay (Figure 5). The treatment of A549 cells with 50 µg/mL of cisplatin for 24 h (Figure 5A) and with 10 µg/mL of cisplatin for 48 h (Figure 5B) resulted in significant cytotoxicity, as indicated by an increased number of dead cells. Conversely, exposure to 1 µM nicotine, 10 µg/mL GNPs, 0.18 µg/mL APS12-2, or 10 µg/mL APS12-2-GNPs (equivalent to 0.18 µg/mL APS12-2) showed no cytotoxic effect at either time interval (24 and 48 h) (Figure 4B and Figure 5A). The cytotoxicity of cisplatin was significantly reduced when the cells were simultaneously co-treated with 1 µM nicotine and cisplatin (Figure 4B and Figure 5A). Although 0.18 µg/mL of APS12-2 alone did not induce cytotoxicity in A549 cells, it significantly restored the cytotoxic effects of cisplatin (Figure 4B and Figure 5A). In contrast, treatment with 10 µg/mL of APS12-2-GNPs did not restore the cytotoxicity of cisplatin in the 24 h treatment (Figure 5A), but both APS12-2 and APS12-2-GNPs restored the cytotoxicity of cisplatin in the 48 h treatment (Figure 5B). At concentrations of 0.18 µg/mL APS12-2 and 10 µg/mL APS12-2-GNPs, no cellular damage was observed in A549 cells (Figure 5C). However, significant cellular damage was evident in cells treated with 50 µg/mL of cisplatin (Figure 5C).

### 3.5. Intracellular ROS Measurement

**Figure 6:**
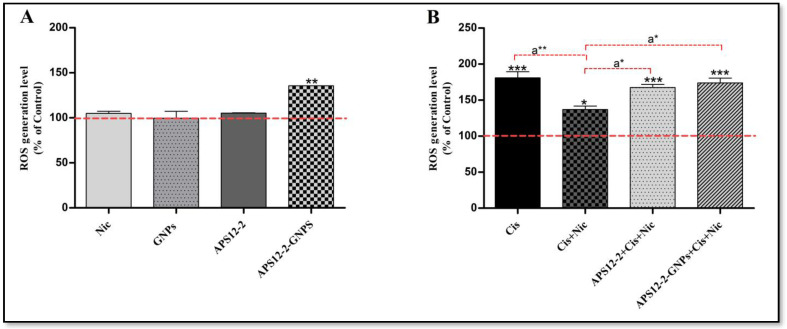
Intracellular ROS formation in A549 cells exposed to different compounds for 24 h. (**A**) ROS levels in cells that were treated with 1 µM nicotine (Nic), 10 µg/mL GNPs, 0.18 µg/mL APS12.2, or 10 µg/mL APS12.2-GNPs (equivalent to 0.18 µg/mL APS12.2). (**B**) ROS levels in cells treated with 100 µg/mL cisplatin (Cis); a combination of Nic and Cis; a combination of APS12.2, Cis, and Nic; or a combination of APS12.2-GNPs, Nic, and Cis. Results were normalized to the untreated control cells (dashed line) and are presented as mean percentage (+SD, n = 3). The data were statistically analyzed using ANOVA with Bonferroni multiple comparisons post-test. The asterisks indicate a significant difference compared to the untreated control. * equals *p* < 0.05; ** equals *p* < 0.01; *** equals *p* < 0.001 and, with respect to cells treated with Cis and Nic, a* equals *p* < 0.05; a** equals *p* < 0.01.

The level of intracellular reactive oxygen species (ROS) was determined in A549 cells after a 24 h treatment with various compounds (Figure 6). Treatment with nicotine, GNPs, and APS12-2 did not increase ROS content in A549 cells, whereas treatment with APS12-2-GNPs significantly increased ROS content in A549 cells (Figure 6A). After the addition of 100 µg/mL cisplatin, ROS levels were significantly increased in A549 cells (Figure 6B). Notably, pretreatment with 1 µM nicotine reduced the ROS induction by cisplatin, compared to cells without nicotine pretreatment (Figure 6B). This effect of nicotine was attenuated by the presence of 0.18 µg/mL APS12-2 and 10 µg/mL APS12-2-GNPs (Figure 6B).

### 3.6. Intracellular Amount of Lipid Droplets

**Figure 7:**
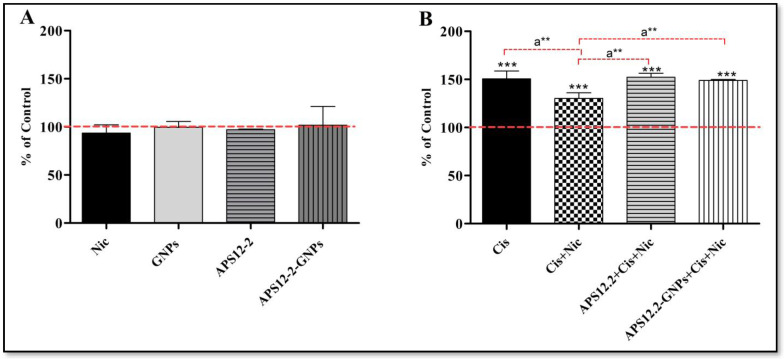
Amount of lipid droplets in A549 cells exposed to different compounds for 24 h. (**A**) Cells were treated with 1 µM nicotine (Nic), 10 µg/mL GNPs, 0.18 µg/mL APS12.2, or 10 µg/mL APS12.2-GNPs (equivalent to 0.18 µg/mL APS12.2). (**B**) 50 µg/mL cisplatin (Cis); a combination of Nic and Cis; a combination of APS12.2, Cis, and Nic; or a combination of APS12.2-GNPs, Nic, and Cis. Measurements were normalized to untreated controls (dashed line) and presented as a mean percentage (+SD, n = 3). The data were statistically analyzed using ANOVA with Bonferroni multiple comparisons post-test. The asterisks indicate a significant difference with respect to the untreated control. *** equals *p* < 0.001 and, with respect to cells treated with Cis and Nic, a** equals *p* < 0.01.

The amount of lipid droplets (LDs) was assessed using Nile red staining in A549 cells, following a 24 h treatment with various compounds (Figure 7). Treatment with 10 µg/mL GNPs, 0.18 µg/mL APS12.2, and 10 µg/mL APS12.2-GNPs did not affect the amount of lipid droplets (LDs) in A549 cells (Figure 7A). However, treatment with 50 µg/mL cisplatin significantly increased the amount of lipid droplets in A549 cells (Figure 7B). Interestingly, the cisplatin-induced increase in LDs was significantly reduced when cells were co-treated with 1 µM nicotine and 50 µg/mL cisplatin (Figure 7B). Conversely, the co-treatment of cells with nicotine, along with APS12-2 or APS12-2-GNPs, restored the cisplatin-induced increase in LDs (Figure 7B).

## 4. Discussion

The use of NPs offers several advantages in various medical applications, from diagnostics to cancer treatment [36,37]. Several types of NPs were developed specifically for targeted drug delivery to treat lung cancer and lung metastases [37]. In our study, GNPs and GNPs loaded with a synthetic analog of marine sponge toxin APS12-2 (APS12-2-GNPs) were prepared using a nanoprecipitation method [32] that allows the production of significantly smaller particles (100–200 nm), in comparison to the coacervation method, where GNPs diameter size range between 200 and 500 nm [38]. The zeta potential of GNPs (−26.0 mV) was notably lower compared to that of APS12-2-GNPs (8.3 mV), as shown in Figure 2. This indicates that APS12-2-GNPs are less stable and more prone to nanoparticle aggregation than free GNPs (Figure 2). Typically, zeta potentials of approximately ±20 mV suggest only short-term stability for nanoparticles, while values below 5 mV are indicative of rapid aggregation [39]. This difference in stability might also explain the higher hydrodynamic diameter of GNPs compared to APS12-2-GNPs.

Subsequently, the cytotoxicity of prepared GNPs and APS12-2-GNPs, as well as APS12-2, on A549 non-small-cell lung cancer and BEAS-2B non-cancer cells was investigated. A549 lung cells were chosen in this study for their high expression level of α7 nAChRs [40]. Our results showed that GNPs were not cytotoxic to both A549 lung cancer cells and BEAS-2B non-cancer cells (Figure 3A,B). The outcome is expected, as GNPs are well-known for their good biodegradability and non-toxic nature, making them promising candidates for drug delivery and controlled release [30]. The cytotoxicity of APS12-2 was reduced in both cell lines, when loaded into GNPs (Figure 4C,D). It is known that various APS molecules, including APS12-2, can form pores in cell membranes, making them cytotoxic [23]. By incorporating APS12-2 into GNPs, we probably reduce the concentration of free APS12-2 that comes into direct contact with the plasmalemma, making APS12-2-GNPs slightly less cytotoxic. GNPs, as nanocarriers, enable slow drug release [36], which, in the case of APS12-2-GNPs, can induce cytotoxicity in cells that have endocytosed APS12-2-GNPs. The cytotoxicity of APS12-2, when loaded in GNPs, was diminished in BEAS-2B cells, but only slightly reduced in A549 cells, at a concentration of 0.45 µg/mL, equivalent to 10 µg/mL APS12-2GNPs (Figure 4C,D). This could be explained by a higher endocytosis of nanoparticles by A549 cells, compared to BEAS-2B cells, which has already been shown in the case of polystyrene nanoparticles [41].

This study aimed to investigate the potential of APS12-2 and APS12-2-GNPs as nAChR antagonists, to reduce the effects of nicotine on the efficacy of cisplatin-based chemotherapy. Nicotine binds to and activates nAChRs, leading to various signaling pathways that increase lung cancer invasiveness and resistance to chemotherapeutics [11]. Our study observed that for the co-treatment of A549 cells with cisplatin and nicotine, nicotine significantly reduced the cytotoxicity of cisplatin (Figure 4A,B), potentially via nAChR activation. Similarly, a previous study observed that the cytotoxicity of cisplatin was significantly decreased by nicotine through α7 nAChRs in oral cancer cells (YD8 and OEC-M1) [11]. Additionally, our previous study demonstrated that nicotine reduced the cytotoxicity of cisplatin on A549 cells [25]. Furthermore, the cytotoxicity of cisplatin was restored by the nAChR antagonist APS12-2 (Figure 4A,B). Similarly, our previous study demonstrated that the α7 nAChR antagonist APS8-2 restored cisplatin-induced cytotoxicity in A549 cells, by blocking the effects of nicotine [25]. APS12-2-GNPs did not restore cisplatin cytotoxicity in the 24 h treatment (Figure 4A), whereas they were able to restore it in the 48 h treatment (Figure 4B). We could speculate that APS12-2-GNPs were unable to restore the cytotoxicity of cisplatin in the 24 h treatment, due to the reduced concentration of free APS12-2, which directly interacts with nAChRs, when APS12-2 was loaded into GNPs. This reduction in free APS12-2 could be attributed to the slower release of APS12-2-GNPs from the nanoparticles, known for enabling slow drug release [36], as illustrated in Figure 3B, which indicated a gradual release of APS12-2 over a 6–48 h period.

nAChRs are expressed not only on the outer cell membranes, but also on the outer membranes of mitochondria [37]. The activation of mitochondrial α7-nAChRs inhibits mitochondrial permeability transition pore (mPTP) opening and cytochrome c release, thereby blocking the early apoptosis stages [9,42,43]. The mitochondrial nAChRs were found to primarily mediate nicotine’s ability to protect SW900 human lung cancer cells against apoptotic agents such as hydrogen peroxide and staurosporine [44]. In our research, nicotine might also decrease the cytotoxicity of cisplatin (Figure 4A,B), potentially via mitochondrial nAChRs. Furthermore, APS12-2, either free or released from APS12-2-GNPs, might attenuate the effects of nicotine on mitochondrial α7-nAChRs, leading to the restoration of the cytotoxicity of cisplatin. On the other hand, our results showed that approximately 70% of APS12-2 was released within 6 h (Figure 3B), indicating that a large amount of APS12-2 might be released from APS12-2-GNPs into the extracellular environment. The released APS12-2 might then be able to block membrane nAChRs, thereby blocking nicotine’s effects on the cytotoxicity of cisplatin and leading to the restoration of cisplatin’s cytotoxicity (Figure 4B).

Considering cisplatin-induced cytotoxicity involves mitochondrial reactive oxygen species (ROS) generation [14], the effects of nicotine on cisplatin-induced ROS production was investigated (Figure 5). In this study, it was observed that nicotine decreased the intracellular amount of ROS production caused by cisplatin in A549 cells (Figure 5B), potentially via nAChR activation. This outcome could be explained by a previous study [45], which showed that the nicotine can reduce oxidative stress by decreasing ROS generation and activating cellular antioxidant defenses, via the activation of α7 nAChRs [45]. Dong et al. (2020) suggested that the activation of α7-nAChRs by nicotine protects against H_2_O_2_-induced oxidative injury, through a signaling pathway that leads to the phosphorylation of Erk1/2 [45]. We speculate that nicotine might decrease cisplatin-induced ROS production (Figure 5B) via the activation of α7 nAChRs, leading to a decrease in the cytotoxicity of cisplatin (Figure 4). Furthermore, this study demonstrated that APS12-2 attenuated the nicotine-induced decrease in ROS generated by cisplatin (Figure 5B). This decrease in ROS generated by cisplatin was also prevented by APS12-2-GNPs, possibly due to the ROS generated from APS12-2-GNPs (Figure 5A). Similarly, APS8-2 showed the potential to attenuate the nicotine-induced decrease in ROS generated by cisplatin [25]. We speculate that APS12-2 might bind to and block α7 nAChRs, thereby attenuating the effects of nicotine on cisplatin. Further studies are needed to explore the binding selectivity of APS12-2 to different sub-types of nAChRs.

Additionally, we observed a correlation between ROS production and lipid droplet (LD) formation. It has already been reported that ROS plays a crucial role in the formation of LDs [46]. LDs are organelles found in the cytoplasm that play a crucial role in storing neutral lipids [47]. In this study, cisplatin significantly increased the amount of LDs in A549 cells (Figure 6B). This increase in the amount of LDs by cisplatin might be due to ROS generated by cisplatin (Figure 5B). However, when A549 cells were treated with cisplatin and nicotine, we observed not only a decrease in intracellular ROS formation (Figure 5B), but also a decrease in the cellular amount of LDs (Figure 6B). This reduction could be attributed to the activation of nAChRs by nicotine. However, when A549 cells were simultaneously treated with nAChR antagonists (APS12-2 or APS12-2-GNPs), nicotine and cisplatin, the nicotine-induced reduction in LDs was attenuated (Figure 6B), potentially via blocking nAChRs.

Our study showed that APS12-2 and APS12-2-GNPs restored the cytotoxic effect of cisplatin on cancer cells. Compared to APS12-2-GNPs, free APS12-2 exhibited a higher cytotoxicity on cancer and non-cancer cells. However, both APS12-2 and APS12-2-GNPs showed the potential to attenuate the nicotine-induced reduction in ROS and LDs caused by cisplatin. These results suggest that APS12-2 and APS12-2-GNPs could be used to enhance the effectiveness of cisplatin treatment for lung cancer. Further in vivo studies are needed to confirm the potential of APS12-2 APS12-2-GNPs to be used as supportive agents in the cisplatin-based chemotherapy of lung cancer.

## 5. Conclusions

Incorporating APS12-2 into GNPs decreases its cytotoxic effects on non-cancerous lung cells, more so than on lung cancer cells. Nicotine reduces cisplatin cytotoxicity and cisplatin induced increases in cellular amounts of ROS and LDs. Our study demonstrates that these effects of nicotine are reduced by treatment with APS12-2 and APS12-2-GNPs. Incorporating APS12-2 into GNPs decreases the potential of APS12-2 to restore the cisplatin cytotoxicity decreased by nicotine in 24 h treatment. Our findings indicate that APS12-2 and APS12-2-GNPs hold promise as supportive agents in the cisplatin-based chemotherapy of lung cancer.

## Data Availability

The data supporting the findings of this study are available from the corresponding author upon request.

## References

[1] Sung H., Ferlay J., Siegel R.L., Laversanne M., Soerjomataram I., Jemal A., Bray F. (2021). Global Cancer Statistics 2020: GLOBOCAN Estimates of Incidence and Mortality Worldwide for 36 Cancers in 185 Countries. CA Cancer J. Clin..

[2] Woodman C., Vundu G., George A., Wilson C.M. (2020). Applications and strategies in nanodiagnosis and nanotherapy in lung cancer. Semin. Cancer Biol..

[3] Kunda N.K. (2020). Antimicrobial peptides as novel therapeutics for non-small cell lung cancer. Drug Discov. Today.

[4] Cho K., Wang X., Nie S., Chen Z.G., Shin D.M. (2008). Therapeutic Nanoparticles for Drug Delivery in Cancer. Clin. Cancer Res..

[5] Araghi M., Mannani R., Maleki A.H., Hamidi A., Rostami S., Safa S.H., Faramarzi F., Khorasani S., Alimohammadi M., Tahmasebi S. (2023). Recent advances in non-small cell lung cancer targeted therapy; an update review. Cancer Cell Int..

[6] Zhang C., Leighl N.B., Wu Y.-L., Zhong W.-Z. (2019). Emerging therapies for non-small cell lung cancer. J. Hematol. Oncol..

[7] Zovko A., Viktorsson K., Lewensohn R., Kološa K., Filipič M., Xing H., Kem W.R., Paleari L., Turk T. (2013). APS8, a Polymeric Alkylpyridinium Salt Blocks α7 nAChR and Induces Apoptosis in Non-Small Cell Lung Carcinoma. Mar. Drugs.

[8] Schuller H.M. (2009). Is cancer triggered by altered signalling of nicotinic acetylcholine receptors?. Nat. Rev. Cancer.

[9] Grando S.A. (2014). Connections of nicotine to cancer. Nat. Rev. Cancer.

[10] Mucchietto V., Fasoli F., Pucci S., Moretti M., Benfante R., Mclntosh M., Clementi F., Gotti C. (2018). A9- and A7-Containing Receptors Mediate the pro-Proliferative Effects of Nicotine in the A549 Adenocarcinoma Cell Line. Br. J. Pharmacol..

[11] Hsu C.-C., Tsai K.-Y., Su Y.-F., Chien C.-Y., Chen Y.-C., Wu Y.-C., Liu S.-Y., Shieh Y.S. (2020). α7-Nicotine acetylcholine receptor mediated nicotine induced cell survival and cisplatin resistance in oral cancer. Arch. Oral Biol..

[12] Schuller H.M. (2012). Regulatory Role of the A7nAChR in Cancer. Curr. Drug Targets.

[13] Chen Y.-J., Wang Z.-W., Lu T.-L., Gomez C.B., Fang H.-W., Wei Y., Tseng C.-L. (2020). The Synergistic Anticancer Effect of Dual Drug- (Cisplatin/Epigallocatechin Gallate) Loaded Gelatin Nanoparticles for Lung Cancer Treatment. J. Nanomater..

[14] Choi Y.-M., Kim H.-K., Shim W., Anwar M.A., Kwon J.-W., Kwon H.-K., Kim H.J., Jeong H., Kim H.M., Hwang D. (2015). Mechanism of Cisplatin-Induced Cytotoxicity Is Correlated to Impaired Metabolism Due to Mitochondrial ROS Generation. PLoS ONE.

[15] Renschler M.F. (2004). The emerging role of reactive oxygen species in cancer therapy. Eur. J. Cancer.

[16] Casares C., Ramírez-Camacho R., Trinidad A., Roldán A., Jorge E., García-Berrocal J.R. (2012). Reactive oxygen species in apoptosis induced by cisplatin: Review of physiopathological mechanisms in animal models. Eur. Arch. Oto-Rhino-Laryngol..

[17] Vasconcellos V.F., Marta G.N., da Silva E.M., Gois A.F., de Castria T.B., Riera R. (2020). Cisplatin versus carboplatin in combination with third-generation drugs for advanced non-small cell lung cancer. Cochrane Database Syst. Rev..

[18] Bai S., Wen W., Hou X., Wu J., Yi L., Zhi Y., Lv Y., Tan X., Liu L., Wang P. (2021). Inhibitory effect of sinomenine on lung cancer cells via negative regulation of α7 nicotinic acetylcholine receptor. J. Leukoc. Biol..

[19] Jiang W., Fan W., Gao T., Li T., Yin Z., Guo H., Wang L., Han Y., Jiang J.-D. (2020). Analgesic Mechanism of Sinomenine against Chronic Pain. Pain Res. Manag..

[20] Witayateeraporn W., Arunrungvichian K., Pothongsrisit S., Doungchawee J., Vajragupta O., Pongrakhananon V. (2020). α7-Nicotinic acetylcholine receptor antagonist QND7 suppresses non-small cell lung cancer cell proliferation and migration via inhibition of Akt/mTOR signaling. Biochem. Biophys. Res. Commun..

[21] Brown K.C., Lau J.K., Dom A.M., Witte T.R., Luo H., Crabtree C.M., Shah Y.H., Shiflett B.S., Marcelo A.J., Proper N.A. (2011). MG624, an α7-nAChR antagonist, inhibits angiogenesis via the Egr-1/FGF2 pathway. Angiogenesis.

[22] Grandič M., Bajuk B.P., Sepčić K., Košorok M.D., Frangež R. (2013). Effects of synthetic analogues of poly-APS on contractile response of porcine coronary arteries. Toxicol. Vitr..

[23] Houssen W.E., Lu Z., Edrada-Ebel R., Chatzi C., Tucker S.J., Sepčić K., Turk T., Zovko A., Shen S., Mancini I. (2010). Chemical synthesis and biological activities of 3-alkyl pyridinium polymeric analogues of marine toxins. J. Chem. Biol..

[24] Zovko A., Gabrič M.V., Sepčić K., Pohleven F., Jaklič D., Gunde-Cimerman N., Lu Z., Edrada-Ebel R., Houssen W.E., Mancini I. (2012). Antifungal and antibacterial activity of 3-alkylpyridinium polymeric analogs of marine toxins. Int. Biodeterior. Biodegrad..

[25] Joukhan A., Kononenko V., Bele T., Dolenc M.S., Peigneur S., Pinheiro-Junior E.L., Tytgat J., Turk T., Križaj I., Drobne D. (2024). Attenuation of Nicotine Effects on A549 Lung Cancer Cells by Synthetic α7 nAChR Antagonists APS7-2 and APS8-2. Mar. Drugs.

[26] Grandič M., Sepčić K., Turk T., Juntes P., Frangež R. (2011). In vivo toxic and lethal cardiovascular effects of a synthetic polymeric 1,3-dodecylpyridinium salt in rodents. Toxicol. Appl. Pharmacol..

[27] Grandič M., Aráoz R., Molgó J., Turk T., Sepčić K., Benoit E., Frangež R. (2012). The non-competitive acetylcholinesterase inhibitor APS12-2 is a potent antagonist of skeletal muscle nicotinic acetylcholine receptors. Toxicol. Appl. Pharmacol..

[28] Abdolmaleki A., Asadi A., Gurushankar K., Karimi Shayan T., Abedi Sarvestani F. (2021). Importance of Nano Medicine and New Drug Therapies for Cancer. Adv. Pharm. Bull..

[29] Wolfram J., Ferrari M. (2019). Clinical cancer nanomedicine. Nano Today.

[30] Yasmin R., Shah M., Khan S.A., Ali R. (2017). Gelatin nanoparticles: A potential candidate for medical applications. Nanotechnol. Rev..

[31] Jiang X., Du Z., Zhang X., Zaman F., Song Z., Guan Y., Yu T., Huang Y. (2023). Gelatin-based anticancer drug delivery nanosystems: A mini review. Front. Bioeng. Biotechnol..

[32] Khan S.A., Schneider M. (2013). Improvement of Nanoprecipitation Technique for Preparation of Gelatin Nanoparticles and Potential Macromolecular Drug Loading. Macromol. Biosci..

[33] Liwata H.N., Liyongo C.I., Mayaliwa J.C.M., Ilunga E.M., Mumba C.M., Mbombo C.M., Muzele T.K. (2020). Phytochemical Screening and Antibacterial Activity of Phytomedecine Mathesia, a Drug Use against Buruli Ulcer in Republic Democratic of the Congo (DRC). Eur. J. Pharm. Med. Res..

[34] Wardini T.H., Afifa I.N., Esyanti R.R., Astutiningsih N.T., Pujisiswanto H. (2023). The potential of invasive species *Praxelis clematidea* extract as a bioherbicide for *Asystasia gangetica*. Biodiversitas J. Biol. Divers..

[35] Kononenko V., Drobne D. (2019). In Vitro Cytotoxicity Evaluation of the Magnéli Phase Titanium Suboxides (Ti_x_O_2x−1_) on A549 Human Lung Cells. Int. J. Mol. Sci..

[36] Carrasco-esteban E., Domínguez-rullán J.A., Barrionuevo-castillo P., Pelari-mici L., López-campos F. (2021). Current Role of Nanoparticles in the Treatment of Lung Cancer. J. Clin. Transl. Res..

[37] Yazdi M.E.T., Qayoomian M., Beigoli S., Boskabady M.H. (2023). Recent advances in nanoparticle applications in respiratory disorders: A review. Front. Pharmacol..

[38] Lee E.J., Khan S.A., Lim K.-H. (2011). Gelatin Nanoparticle Preparation by Nanoprecipitation. J. Biomater. Sci. Polym. Ed..

[39] Németh Z., Csóka I., Jazani R.S., Sipos B., Haspel H., Kozma G., Kónya Z., Dobó D.G. (2022). Quality by Design-Driven Zeta Potential Optimisation Study of Liposomes with Charge Imparting Membrane Additives. Pharmaceutics.

[40] Mei D., Zhao L., Chen B., Zhang X., Wang X., Yu Z., Ni X., Zhang Q. (2018). α-Conotoxin ImI-modified polymeric micelles as potential nanocarriers for targeted docetaxel delivery to α7-nAChR overexpressed non-small cell lung cancer. Drug Deliv..

[41] Liu Y.-Y., Liu J., Wu H., Zhang Q., Tang X.-R., Li D., Li C.-S., Liu Y., Cao A., Wang H. (2023). Endocytosis, Distribution, and Exocytosis of Polystyrene Nanoparticles in Human Lung Cells. Nanomaterials.

[42] Chernyavsky A.I., Shchepotin I.B., Galitovkiy V., Grando S.A. (2015). Mechanisms of tumor-promoting activities of nicotine in lung cancer: Synergistic effects of cell membrane and mitochondrial nicotinic acetylcholine receptors. BMC Cancer.

[43] Skok M., Gergalova G., Lykhmus O., Kalashnyk O., Koval L., Uspenska K. (2016). Nicotinic acetylcholine receptors in mitochondria: Subunit composition, function and signaling. Neurotransmitter.

[44] Friedman J.R., Richbart S.D., Merritt J.C., Brown K.C., Nolan N.A., Akers A.T., Lau J.K., Robateau Z.R., Miles S.L., Dasgupta P. (2018). Acetylcholine signaling system in progression of lung cancers. Pharmacol. Ther..

[45] Dong Y., Bi W., Zheng K., Zhu E., Wang S., Xiong Y., Chang J., Jiang J., Liu B., Lu Z. (2020). Nicotine Prevents Oxidative Stress-Induced Hippocampal Neuronal Injury through A7-NAChR/Erk1/2 Signaling Pathway. Front. Mol. Neurosci..

[46] Jin Y., Tan Y., Chen L., Liu Y., Ren Z. (2018). Reactive Oxygen Species Induces Lipid Droplet Accumulation in HepG2 Cells by Increasing Perilipin 2 Expression. Int. J. Mol. Sci..

[47] Welte M.A., Gould A.P. (2017). Lipid droplet functions beyond energy storage. Biochim. Biophys. Acta Mol. Cell Biol. Lipids.

